# Preparation of sulfonated carbon-based catalysts from murumuru kernel shell and their performance in the esterification reaction†

**DOI:** 10.1039/d0ra03217d

**Published:** 2020-05-27

**Authors:** Ana Paula da Luz Corrêa, Rafael Roberto Cardoso Bastos, Geraldo Narciso da Rocha Filho, José Roberto Zamian, Leyvison Rafael Vieira da Conceição

**Affiliations:** Institute of Exact and Natural Sciences, Graduate in Chemistry Program, Laboratory of Catalysis and Oleochemical, Federal University of Pará Belém Pará CEP 66075-110 Brazil rafaelvieira@ufpa.br

## Abstract

In the present study, heterogeneous acid catalysts for fatty acid esterification reactions were synthesized using agro-industrial waste from murumuru kernel shells. The waste was carbonized and functionalized with concentrated sulfuric acid under different sulfonation conditions, obtaining the sulfonated biochar. The results indicate that the best sulfonation conditions were obtained with a contact time of 4 h, the temperature of 200 °C, and a solid-acid ratio of 1 : 10 (w/v). The best catalyst was characterized by acid–base titration for the determination of total acid density, X-ray diffraction, scanning electron microscopy, X-ray energy dispersion spectroscopy, Fourier transform infrared spectroscopy and thermal analysis. Reaction conditions of oleic acid with methanol and the viability of catalyst reuse were also investigated. A conversion of 97.2% was achieved under optimum esterification reaction conditions, employing 5% catalyst, 10 : 1 molar ratio of methanol to oleic acid, during 1.5 h at a temperature of 90 °C. After 4 reaction cycles, the catalyst preserved its efficiency at 66.3% conversion. The catalyst activity was evaluated in reactions using palmitic acid, soybean fatty acid distillate, palm fatty acid distillate, and coconut fatty acid distillate. The results demonstrate that the catalyst is applicable and efficient in esterification reactions of raw materials, containing different fatty acid compositions since different carbonized materials have varying abilities to combine acid groups. This work reveals the promising feasibility of using biomass generated in large quantities by the agroindustry for the development of a new heterogeneous acid catalyst for biodiesel production.

## Abstract 2

In the present study, heterogeneous acid catalysts for fatty acid esterification reactions were synthesized using agro-industrial waste from murumuru kernel shells.

## Graphical abstract

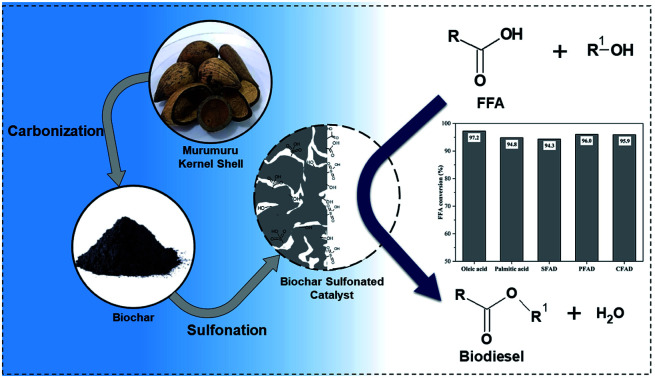

## 1. Introduction

Limited oil reservoirs and greenhouse gas emissions are major problems related to the use of fossil fuels. These factors motivate the search for alternative sources of energy.^1,2^ In this context, biodiesel has attracted great attention as a promising alternative to diesel in recent years. Biodiesel is derived mainly from vegetable oils, animal fat or food oil residues, and has desirable features, such as non-toxicity and biodegradability.^3^ Compared to petroleum-derived diesel, it has similar physicochemical properties and shows a favorable combustion emission profile.^4^ In addition, biodiesel can be used directly or blended with petroleum diesel.^5^

The major economic constraint in biodiesel production is the high cost of raw materials.^6,7^ Thus, acid oils, such as non-edible vegetable oils, waste cooking oils, and others, are viewed as promising raw materials because they are cheaper.^8^ Furthermore, waste oils offer several advantages: they do not compete with the food market; the process recycles the waste oils and reduces production costs, increasing the economic competitiveness of biodiesel.^9,10^

The transesterification reaction carried out with homogeneous alkaline catalysts is the commonly used technology in the biodiesel industry. However, when oils with high acid values are used with basic catalysts, the production efficiency lowers due to saponification reactions.^11^ The use of traditional homogeneous acid catalysts has some disadvantages, such as undesirable side reactions, equipment corrosion and a large amount of wastewater for treatment, increasing the environmental pollution. Thus, heterogeneous acid catalysts are a better alternative for transesterification reactions when oils with high acidity are used. Besides, they have the advantage of being non-corrosive and can be easily recovered and reused.^12^

Several types of heterogeneous acid catalysts have been investigated, including zeolites,^13^ sulfated zirconia,^14^ anion exchange resin,^15^ and heteropoly acids.^16^ However, most report problems like low stability, low acid density, and stringent reaction conditions. Contrarily, carbon-based catalysts have advantages such as low preparation cost and high catalytic performance. Also, they are relatively cheap, widely available and easily functionalized, making them interesting catalysts in the esterification reaction for biodiesel production.^5,12^

Materials such as oilseed cake, starch, bagasse, and other biomass have been used as carbon precursors for the preparation of heterogeneous acid catalysts.^17^ The kind of carbonaceous material derived from biomass residues is denoted as biochar, which results from an incomplete carbonization process of the initial raw material. Biochar has an abundant oxygen content (27–34% by mass), mostly in the form of phenolic and carboxylic acid groups, with traces of nitrogen and sulfur. Sulfonated biochar containing –SO_3_H groups is the most reported in the literature.^18–20^

An interesting phenomenon was found when comparing the experimental results of different sulfonated biochar catalysts in the esterification and transesterification reactions: different carbonized materials have distinct abilities for the combination of acid groups –SO_3_H.^21^ The existing literature indicates that only a small amount of the biomass involved in the preparation of heterogeneous catalysts for biodiesel production has been studied so far when compared to other chemicals used. Thus, the research should focus on incorporating more biomass waste and decreasing as much as possible the use of conventional chemicals.^6^

Murumuru (*Astrocaryum murumuru* Mart.) is a palm of medium height. These palm trees grow preferably in lowland soils in the Amazon region, and the stone fruit consists of a woody shell.^22^ The oil extracted from murumuru kernels is a semi-solid fat called murumuru butter, which can be used in the production of margarine, in the cosmetic industry for the manufacture of soaps, creams, shampoos, and in the paint industry as a drying agent. As for the kernel shell, it remains a vastly unexplored agro-industrial residue.^23^ Studies on the utilization of this agro-industrial waste are scarce in the literature. Consequently, the present work aims to synthesize an acid catalyst obtained from the murumuru kernel shell, as well as to investigate the sulfonation conditions of the biochar from this waste, and their application in the esterification reaction for biodiesel production.

## 2. Materials and methods

### 2.1. Materials

The murumuru kernel shell (*Astrocaryum murumuru* Mart.) was obtained from the vegetable oil processing company Beraca Ingredientes Naturais Ltd., located in Ananindeua (Pará, Brazil). In the sulfonation process, sulfuric acid 98% (Êxodo®, 7664-93-9) was used. For the esterification reactions, methanol 99.8% (Nuclear®, 67-56-1), oleic acid 99% (Impex®, 112-80-1), palmitic acid 98% (Vetec®, 57-10-3), soybean fatty acid distillate, and coconut fatty acid distillate obtained from local commerce were used, as well as palm fatty acid distillate obtained from the vegetable oils and derivatives company Agropalma Ltd., located in Belém (Pará, Brazil). To measure the yield of methyl esters, methyl heptadecanoate 99% (Sigma-Aldrich®, 1731-92-6) and heptane 95% (Dinâmica®, 142-82-5) were used. Ethanol 99.8% (Êxodo®, 64-17-5) was used in the catalyst recovery (washing) process. All reagents used were of analytical grade.

### 2.2. Catalyst synthesis

**Fig. 1:**
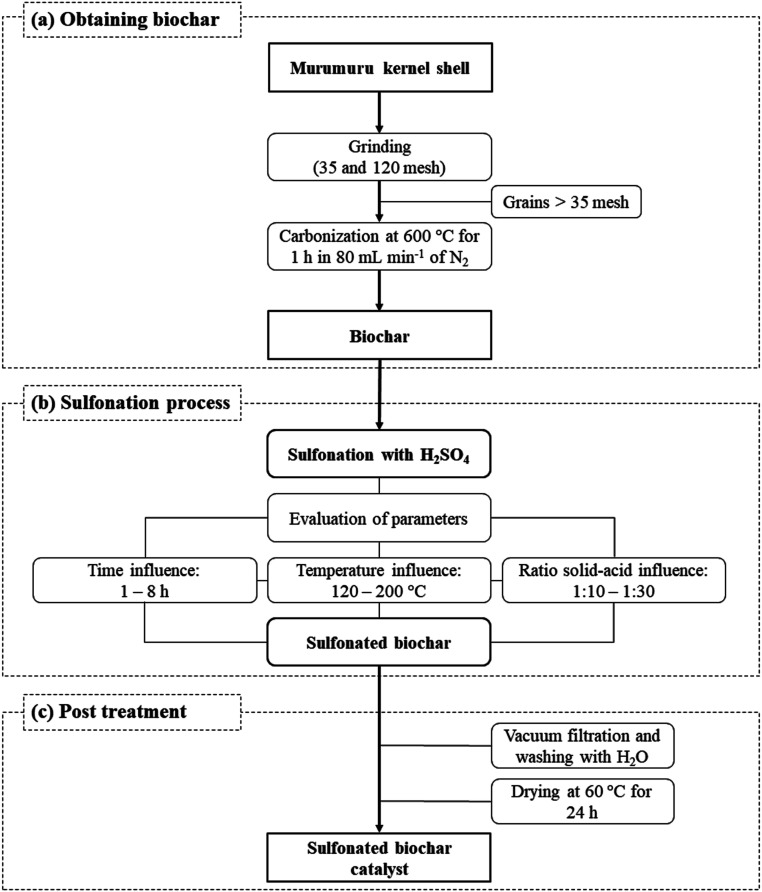
Scheme of catalyst synthesis process.

The catalyst preparation process consists basically of three steps, as illustrated in Fig. 1. The murumuru kernel shell was used as raw material for the preparation of the biochar, subjected initially to a grinding process, followed by grading using sieves of 35 and 120 mesh. The shell grains fraction with greater retention (>35 mesh) were carbonized in a tubular oven at 600 °C for 1 h, with nitrogen flow of 80 mL min^−1^, following a methodology adapted from Bora *et al.*^8^

The obtained biochar was sulfonated in a flat-bottomed flask connected to a condenser and hot plate under constant agitation and different conditions of time, temperature and solid-acid reason (w/v, in g of biochar per mL of concentrated H_2_SO_4_), to evaluate the impact of these variables on the sulfonation process. The time variable was studied in the range of 1–8 h, at a temperature of 200 °C, and a solid-acid ratio of 1 : 10 (w/v). The effect of the temperature was investigated between 120 and 200 °C, with a sulfonation time of 4 h, and a solid-acid ratio of 1 : 10 (w/v). The solid-acid ratio was evaluated in the range between 1 : 10–1 : 30 (w/v), at a temperature of 200 °C and 4 h.

After the sulfonation step, the solids were vacuum filtered, washed with deionized water until neutral pH, and dried in an oven at 60 °C for 24 h to obtain the sulfonated biochar.

### 2.3. Catalyst characterization

The total acid density of the catalyst was determined by acid–base back titration, according to the method adapted from Boehm,^24^ where 0.1 g of catalyst was dispersed in 20 mL of a NaOH 0.1 mol L^−1^ solution and kept under constant agitation for 1 h. Then, the mixture was centrifuged, the supernatant removed and titrated with a HCl 0.1 mol L^−1^ solution, using phenolphthalein as an indicator. The catalyst powder X-ray diffraction (XRD) patterns were collected using a PANalytical diffractometer model EMPYREAN, with applied radiation of Cu Kα (1.54 Å) at 40 kV and 30 mA scanning interval 8°〈2*θ*〉70°. The catalyst morphology was recorded by scanning electron microscopy (SEM), using a Tescan microscope, model VEGA 3 LMU. Elemental surface analysis of the catalyst was determined by X-ray energy dispersion spectroscopy (EDS), using an equipment of microanalysis system, model AZTec Energy X-act with 129 eV resolution and Oxford brand. The functional groups presented in the catalyst were analyzed by Fourier-transform infrared spectroscopy (FT-IR) using a PerkinElmer spectrometer, Spectrum Two model, with a ZnSe crystal horizontal attenuated total reflectance (HATR) accessory. The spectra were collected in the region from 4000 to 400 cm^−1^ with a resolution of 4 cm^−1^. Thermal gravimetric analysis (TGA) was performed in a temperature range of 25 °C to 1000 °C, in a platinum crucible, at a heating rate of 10 °C min^−1^ under a nitrogen atmosphere with a flow rate of 50 mL min^−1^. The equipment was a Shimadzu thermogravimetric analyzer-model DTG-60H.

### 2.4. Esterification reactions

Catalytic tests were performed through esterification reactions of oleic acid with methanol, using sulfonated biochar obtained from the different sulfonation conditions. Esterification reactions were also performed in the presence of only the carbon (support) and in the reaction system without the catalyst (oleic acid + methanol) to evaluate the effect of the catalyst in the reaction system. The experiments were conducted in a Par Multiple Reactor System-model 500.

The reaction products were centrifuged for catalyst recovery, transferred to a decantation funnel and washed with portions of distilled water heated up to 90 °C, for the removal of water and residual alcohol. Finally, the samples were dried in an oven at 80 °C for 24 h. Later, the resulting biodiesel samples were stored in vials and preserved for further analysis.

The reaction conversion was determined by evaluating the acidity indices between esterification products and the raw material used. The acid value was determined by titration, according to AOCS Cd 3d-63 standard, and the conversion of free fatty acids (FFA) was determined using eqn (1):1
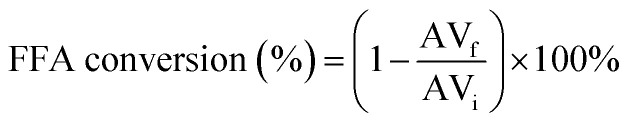
where AV_i_ is the initial acid value (fatty acid) and AV_f_ is the final acid value after the fatty acid esterification reaction, given in mg KOH per g.

The esterification parameters were optimized by assessing the reaction time (0.5–2.5 h), catalyst mass percentage (1–5%), the molar ratio of methanol to oleic acid (5 : 1–25 : 1), and the reaction temperature (60–120 °C).

The catalyst was tested in esterification reactions with different raw materials – palmitic acid, soybean fatty acid distilled (SFAD), palm fatty acid distillate (PFAD) and coconut fatty acid distilled (CFAD) – to verify the catalyst performance in optimized reaction conditions against different fatty acid compositions. The fatty acid composition of these raw materials was determined according to the official methods AOCS Ce 2-66 (preparation of fatty acid methyl esters) and AOCS Ce 1-62 (determination of fatty acid composition). The fatty acid profiles are shown in Table S1, and the chromatograms are illustrated in Fig. S1 (ESI†).

The methyl ester content was determined by gas chromatography (GC), following EN 14103, for biodiesel samples obtained from oleic and palmitic acids, SFAD, PFAD, and CFAD. The chromatograms are presented in Fig. S2 (ESI†).

### 2.5. Catalyst reuse

The reuse test was conducted to determine the number of cycles that the catalyst can be used without requiring regeneration. Thus, 4 reaction cycles of the best performing catalyst were completed maintaining the esterification reaction conditions. To this end, after the reaction and centrifugation processes, the catalyst was separated from the products, washed with ethanol (99.8%), filtered, dried at 60 °C for 24 h, and packaged for later use.

## 3. Results and discussion

### 3.1. Influence of the sulfonation conditions

**Fig. 2:**
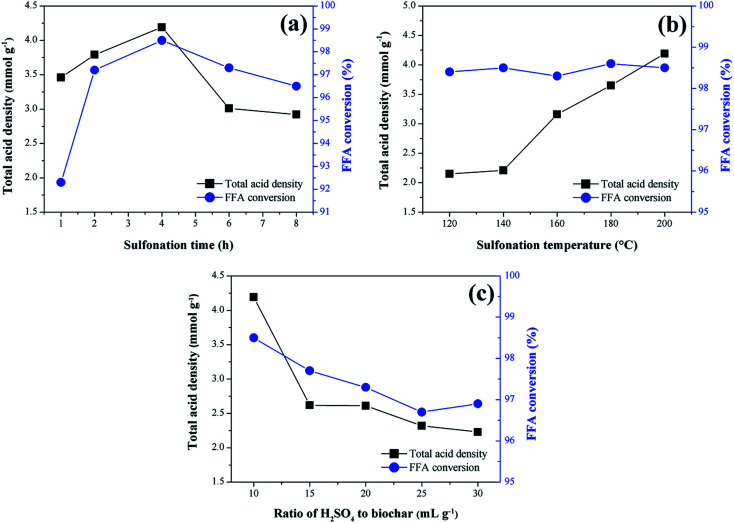
Influence of sulfonation conditions on the catalytic activity of the sulfonated biochar catalyst (esterification at 90 °C for 2 h with the molar ratio of methanol to oleic acid of 20 : 1 and catalyst load of 5 wt%). (a) Sulfonation time (at 200 °C and solid-acid ratio of 1 : 10); (b) sulfonation temperature and (at 4 h and solid-acid ratio of 1 : 10); (c) solid-acid ratio of H_2_SO_4_ to biochar (at 4 h and 200 °C).

The results concerning the influence of the sulfonation variables time, temperature and solid-acid ratio on the catalyst synthesis are shown in Fig. 2. As a response to the catalytic activity, the total acid density values of the surface and the conversion of FFA in the catalysts esterification reactions are evaluated.

From the analysis of Fig. 2a, it is observed that the functionalization of the biochar is partially dependent on the sulfonation time, which can be better understood by analyzing the total acid density values of the catalysts. The presence of the acid sites in the catalysts is certainly a result of the sulfonation process, considering that the acid density determined for non-sulfonated biochar was relatively low, 0.14 mmol g^−1^. It is observed an increase of total acid density values with sulfonation time up to 4 h (4.2 mmol g^−1^), followed by a gradual decrease down to 2.9 mmol g^−1^ at a sulfonation time of 8 h. This result suggests a possible saturation of the functionalization process and consequent degradation of the pyrolyzed material after 4 h of sulfonation. Nevertheless, all sulfonated catalysts showed good catalytic activity during the esterification reactions.

The best FFA conversion results are in the range of 2–6 h sulfonation time, with slight accentuation observed at 4 h (98.5% FFA conversion, and highest total acid density value of 4.2 mmol g^−1^). This suggests a saturation of the reaction catalysis under the studied conditions because the FFA conversion results are not significantly influenced by the sulfonation process, even with the acid density variation. It is noteworthy that similar results were found in studies of carbon-based catalysts prepared from corn straw and glucose–starch mixture, in which the optimum sulfonation process times reported were 4 h and 5 h, respectively.^25,26^

Generally, for biomass sulfonation processes, the functionalization of the material increases with the temperature.^27^ This behavior is also evidenced in the present study because the total acid density values increase with the sulfonation temperature, as Fig. 2b shows. According to Niu *et al.*,^17^ the increase of the temperature may promote the mass transfer between the acid and the biochar to consolidate the sulfonation. The studies conducted by Ngaosuwan, Goodwin and Prasertdham,^28^ which prepared a carbon-based heterogeneous acid catalyst from residues of coffee, indicated an ideal temperature for the sulfonation process similar to the present work, that is 200 °C. However, as observed in the study of the time variable, all sulfonated biochars exhibited high catalytic activity in esterification reactions, proving that the increase of the active sites, after a certain point, will not significantly change the FFA conversion during the first use of the catalyst.

In the solid-acid ratio study, it is observed that from the ratio 1 : 10 (w/v), the increase of sulfuric acid does not influence positively the biochar sulfonation (Fig. 2c), since there is a decrease in the acid density values followed by gradual degradation of the biochar. Therefore, it can be considered that this point approaches the saturated state for functionalization. Studies with different biomass materials such as cassava bark, coconut shell, sawdust, and oleaginous seed cake^29,30^ address a ratio of 1 : 10 (w/v) as the most common in biochar sulfonation processes. However, as seen for the other variables investigated, all catalysts analyzed in this study showed high FFA conversion rates in the esterification reactions.

Generally, sulfonation processes introduce not only sulfonic acid sites but also oxygenated acid sites. According to Zhang *et al.*,^31^ –SO_3_H groups were identified as the primary catalytic active sites, while the carboxyl groups enhanced the inherent activity of –SO_3_H, thus facilitating the esterification.

In the esterification reaction mechanism, the strong acid nature of –SO_3_H group makes the protonation of methanol molecule difficult. However, when the weak acid group such as –COOH is added, the deprotonated form of –COOH can generate hydrogen bonding with the –OH group in the methanol molecule, providing a small portion of “negative charge” to the oxygen in the methanol molecule. In turn, this negative charge promotes the nucleophilicity of the methanol molecule and thus positively influencing the esterification reaction rate and conversion.^31,32^

The esterification reaction of oleic acid with methanol without catalyst showed a conversion of 6.8%, while the esterification reaction in the presence of non-sulfonated biochar reached 7.3%. This shows that non-sulfonated biochar is unable to promote high conversions of FFA in the esterification reaction, and highlights the effectiveness of the sulfonation process by making sulfonated biochar highly active. Thus, the catalyst with the highest total acid density found in the study (4.2 mmol g^−1^) was selected for further investigations due to the tendency of catalysts with higher acid force densities to have better catalytic performance in biodiesel synthesis.^33^ Thus, the sulfonation process variables for the selected catalyst involve a time of 4 h, a temperature of 200 °C, and a solid-acid ratio of 1 : 10 (w/v).

The selected values for the sulfonation process conditions for the production of biochar from murumuru kernel shells are considered optimal when compared with data reported in the literature for other sulfonated biomass. Lathiya, Bhatt and Maheria^34^ studied the preparation of a sulfonated carbon catalyst from orange peel residues. This sulfonation process involved the use of a solid-acid ratio of 1 : 20 w/v biochar to H_2_SO_4_, and 24 h of sulfonation time at 200 °C, obtaining 91.7% of conversion in the esterification reaction using corn acid oil. While Bora *et al.*^8^ synthesized sulfonated activated carbon catalyst from *Mesua ferrea* seed shell residues, under the sulfonation conditions of 1 : 8 w/v carbon to H_2_SO_4_, at 120 °C for 10 h, the maximum conversion obtained in the oil here studied was 95.6%.

### 3.2. Catalyst characterization

**Fig. 3:**
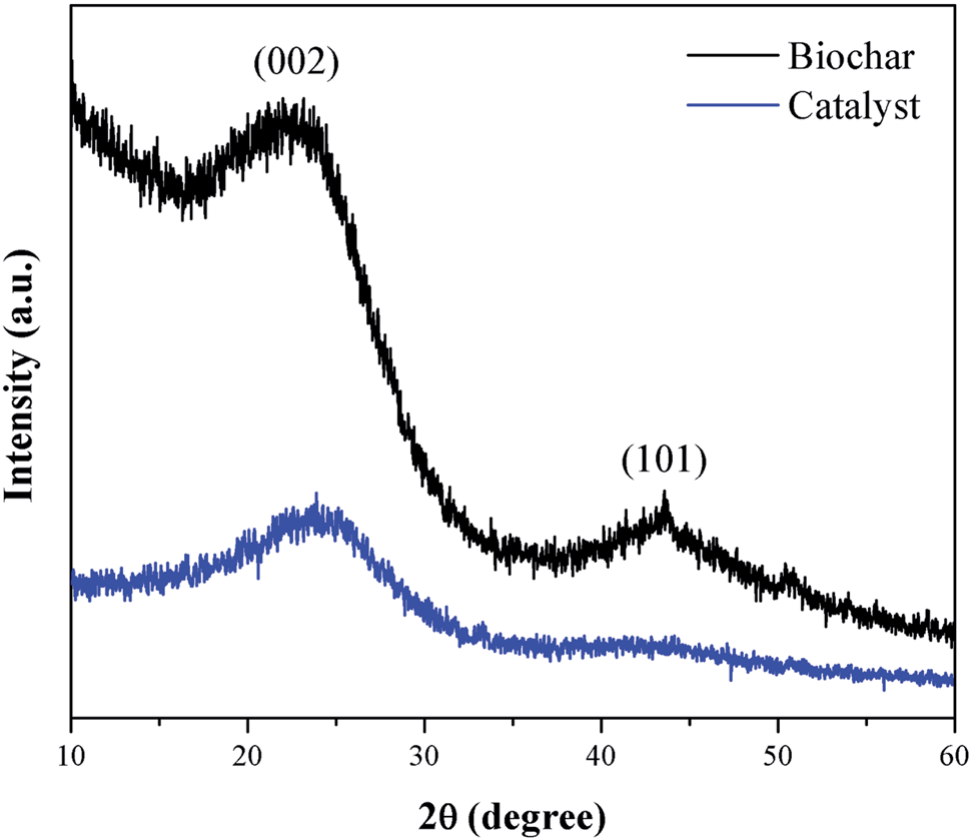
XRD diffraction patterns of biochar and catalyst.

Fig. 3 presents the XRD pattern of the murumuru biochar and the catalyst. The presence of broad diffraction peaks at 2*θ* = 15–30° is attributed to C (002) of amorphous carbon structures containing randomly oriented aromatic carbon sheets. The less intense and wide peaks at 2*θ* = 40–50° are related to C (101) of a graphite structure. After sulfonation, the diffraction peaks become less intense, and slightly shifted to the right, meaning that this process weakens the carbon sheets due to bond breakage, increasing carbonaceous structure disorder.^35–37^

**Fig. 4:**
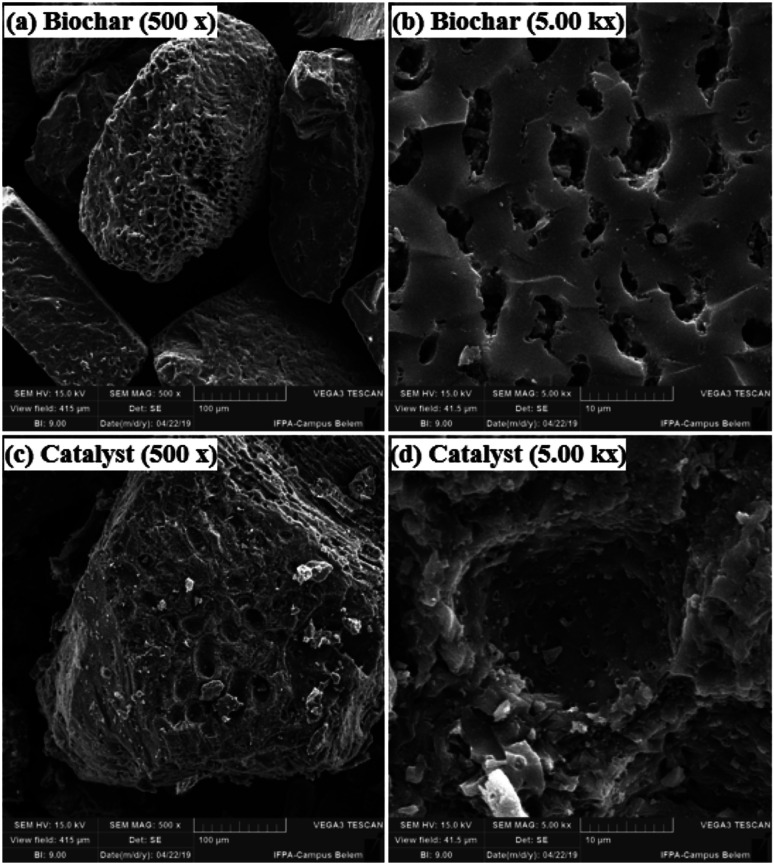
SEM micrographs of: biochar (a) 500× and (b) 5.00k×. Catalyst: (c) 500× and (d) 5.00k×.

Fig. 4 shows the SEM micrographs of the non-sulfonated biochar and the best performing catalyst, demonstrating in detail the surface morphology of these materials. The biochar appearing in Fig. 4a and b exhibits an irregular and heterogeneous surface morphology with a well-developed and accented porous structure, characteristic of carbonized organic waste.^38^ The SEM micrographs of the catalyst surface morphology, shown in Fig. 4c and d, indicate a porosity decrease. This fact can be attributed to occasional small cracks, partial oxidation, condensation and partial destruction of the porous structure, arising with the strong sulfonating agent after the biochar functionalization process.^28,39^ Also, it can be inferred from the micrograph analysis that the partial pore blockage occurs due to the adsorption of –SO_3_H groups on the catalyst support (biochar), confirming the efficiency of the biochar sulfonation process.^8^

**Fig. 5:**
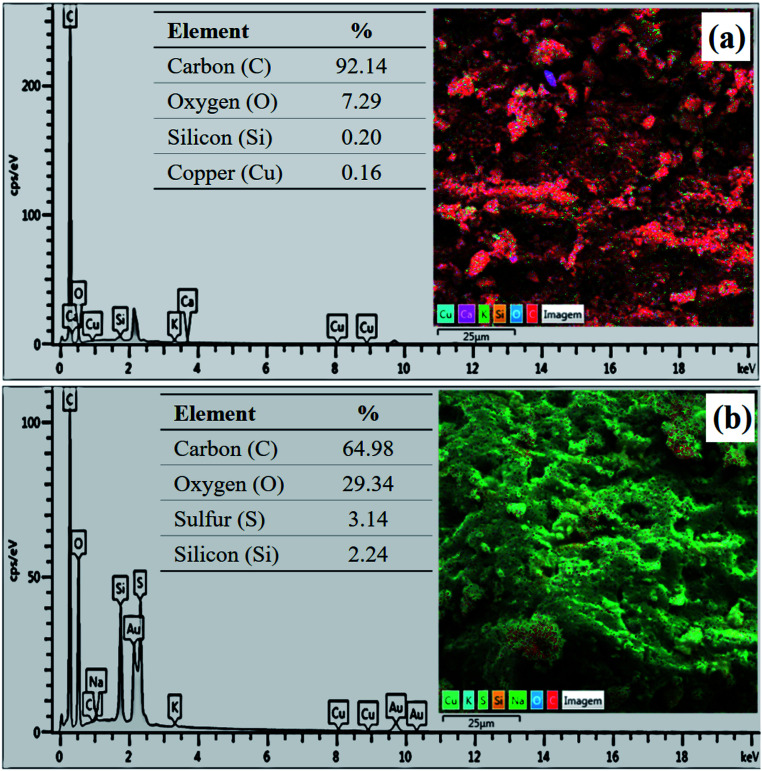
EDS analysis of (a) biochar and (b) catalyst.

The composition determined by EDS analysis reveals a major content of carbon in both materials (Fig. 5), which is due to the pyrolysis process. However, the sample has a content of 3.14% of sulfur after the sulfonation process (Fig. 5b), evidencing the incorporation efficiency of sulfonic groups. The increase in oxygen content of the biochar after functionalization (from 7.29% to 29.34%) shows that the sulfonation with concentrated sulfuric acid also favors the formation of oxygenated groups.

**Fig. 6:**
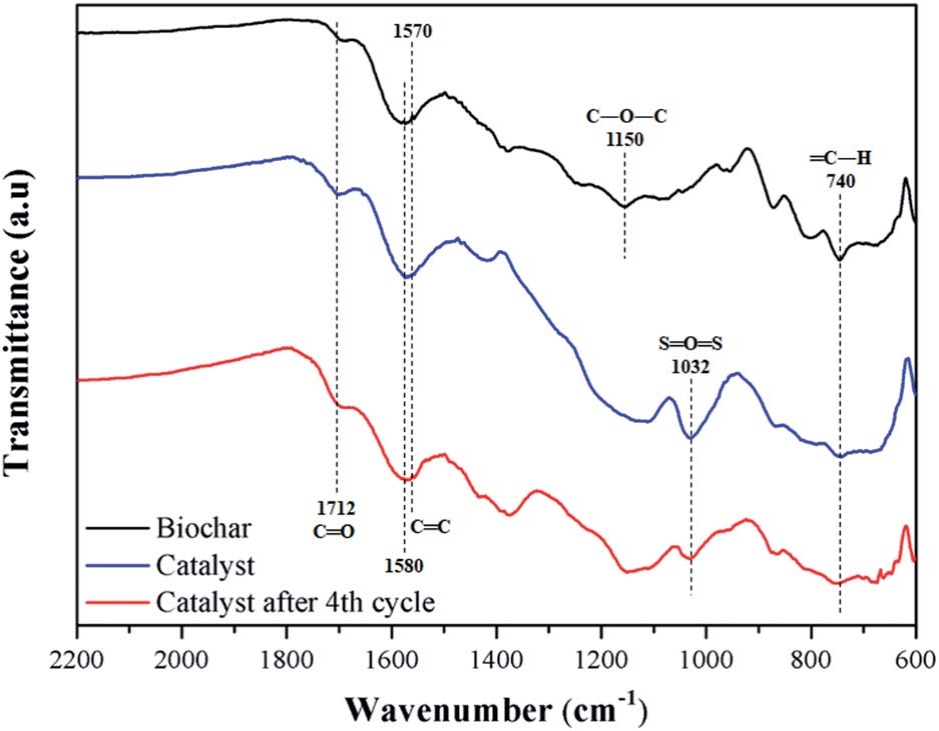
FT-IR spectra of biochar, catalyst and reused catalyst.

Fig. 6 shows the FT-IR spectra of biochar, catalyst, and catalyst after 4 reaction cycles. All samples have typical bands of carbonized material from lignocellulosic raw material, such as C=C, with stretch absorption at 1570 and 1580 cm^−1^, assigned to aromatic rings, and C=O, with stretch bands at 1712 cm^−1^ of carboxylic groups.^40^

For the biochar, stretch bands at 740 cm^−1^ and 1150 cm^−1^ corresponding to bonds type =C–H and C–O–C are observed, respectively.^41^ The catalyst presents the characteristic bands of sulfonated biochar, with emphasis on the 1032 cm^−1^, typically assigned to O=S=O stretching of the sulfonic groups. Also, a slight increase in the intensity of bands C=O stretching at 1712 cm^−1^ is observed, meaning that the sulfonation process favors the formation of carboxylic groups on the material surface.^27^ These results are in agreement with the EDS analysis, where oxygen content increases. After reuse, the intensity of O=S=O stretching type of the catalyst decreased, which is to be expected after several cycles due to the acid group leaching.^34^

**Fig. 7:**
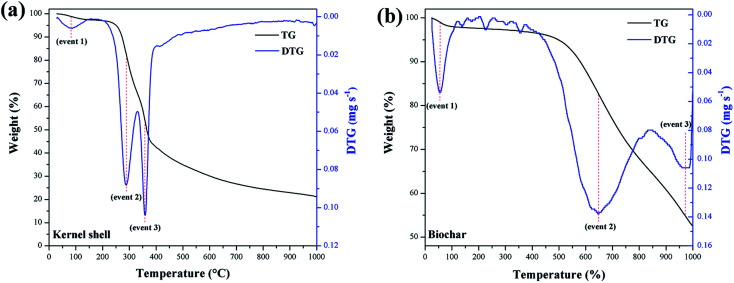
TGA/DTG curves of (a) kernel shell and (b) biochar.

Fig. 7 summarizes the TGA of the materials. The calculation of the first derivative of the curve (DTG) yields the mass loss peaks. The materials presented the first mass loss around 100 °C (event 1) due to the evaporation of water at ambient humidity.^34^ For the murumuru kernel shell (Fig. 7a), mass losses in the temperature range of 168–307 °C (event 2) and between 330–390 °C (event 3) are associated with cellulose, hemicellulose, and lignin degradation.^42^ For biochar (Fig. 7b), mass losses at 565–741 °C (event 2) and between 914–997 °C (event 3) are related to carbon structure decomposition.^43^

**Fig. 8:**
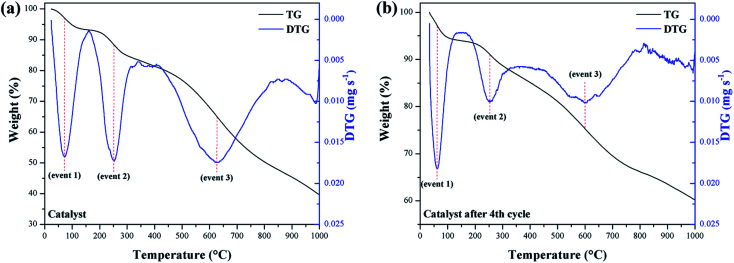
TGA/DTG curves of (a) catalyst and (b) catalyst reused.

In the evaluation of the catalyst stability and its reuse resulting from the fourth reaction cycle, the occurrence of 3 significant mass loss events was observed. TGA/DTG curves for these events are illustrated in Fig. 8a and b, respectively.

Mass losses between 210–290 °C (event 2) are interpreted as a result of the decomposition of the catalyst surface sulfonic groups.^29^ Thus, the catalyst mass loss (9.6%) is observed to be relatively greater than the reused catalyst (8.3%). However, the 8.3% mass loss value for the reused catalyst suggests that the sulfonic groups largely remained on the material surface, even after the fourth reaction cycle. Mass losses at 500–730 °C (event 3) correspond to the decomposition of oxygenated functional groups that may have been introduced as a result of the sulfonation process.^33^ Thus, the mass loss for the catalyst of 36.3%, was higher than for the reused catalyst, which showed a loss of 19.7%.

A possible explanation would be that the –COOH and phenolic –OH groups have a strong affinity between the hydrophilic parts of the reactants and these near neutral carbon-surface groups, which favors interaction with the methanol of the reaction.^44,45^ Thus, such groups also contribute to the catalyst catalytic activity. Consequently, it can be inferred that after the fourth reuse cycle, some of the –COOH and phenolic –OH groups are likely to be leached or degraded during the reactions to which they are subject. As noted from the TGA, the catalyst remained stable under the esterification reaction conditions employed, even after reuse.

### 3.3. Influence of esterification parameters

**Fig. 9:**
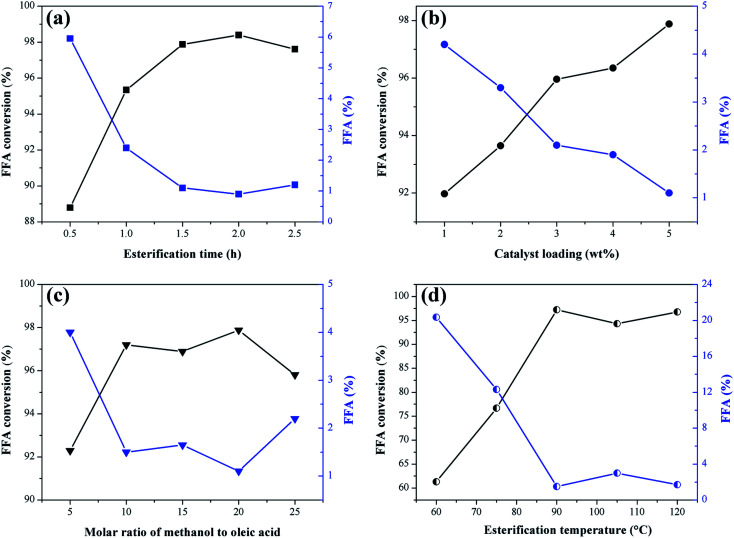
Influence of esterification parameters on catalytic activity of the sulfonated biochar catalyst. (a) Esterification time (at 90 °C with the molar ratio of methanol to oleic acid of 20 : 1 and catalyst load of 5 wt%); (b) catalyst load (at 90 °C for 1.5 h with the molar ratio of methanol to oleic acid of 20 : 1), (c) methanol/oleic acid molar ratio and (d) esterification temperature (for 1.5 h with the molar ratio of methanol to oleic acid of 20 : 1 and catalyst load of 5 wt%).

The effect of the reaction time, catalyst mass percentage, methanol to oleic acid molar ratio and temperature on the esterification reactions are shown in Fig. 9. The response to catalytic activity is given in FFA conversion and the percentage of fatty acids remaining in the product.

It is observed in Fig. 9a that the increase of the reaction time in the interval investigated favors the esterification reaction since there is an increase in the FFA conversion. The values start from 0.5 h, where the conversion is 88.7%, up to 1.5 h, with the conversion of 97.9%. According to Ning and Niu,^5^ the esterification reaction requires sufficient time to ensure mass transfer between the system and once the equilibrium state is reached, the excess time is unnecessary. Consequently, by extending the time up to 2.5 h, there was no significant change in conversion values compared to the obtained at 1.5 h. Therefore, this is the ideal reaction time within the range studied for further investigation of other variables.

The effect of catalyst mass percentage is shown in Fig. 9b. The increase in catalyst percentage in the range investigated causes an increase in FFA conversion values, starting from 1% (w/w) catalyst with 91.9% of conversion, up to 5% (w/w) with 97.9% of conversion. Catalysts are known for providing active sites to facilitate the reaction, so adding catalyst in bulk causes conversion rates to increase. However, the excess of catalyst can increase the viscosity of the reaction medium, difficulting the mass transfer between the catalyst and reagents.^46^ Thus, the optimum amount of catalyst selected was 5% (w/w) for subsequent optimization studies.

In theory, the esterification reaction requires one mole of FFA for one mole of alcohol and the reaction is reversible. However, too much alcohol is required to drive the reaction towards the ester formation.^47,48^ As shown in Fig. 9c, where the molar ratio of methanol to oleic acid ranges from 5 : 1 to 25 : 1, FFA conversion increased from 92.3% at a ratio of 5 : 1 to 97.2% at 10 : 1. From the 10 : 1 molar ratio, the addition of methanol did not provide significant variation in conversion rates. Contrarily, when too much methanol is used, hydrolysis of the ester formed (biodiesel) can occur, causing the reaction to move in the opposite direction.^40^ Excess methanol also dilutes the system, reducing the contact between catalysts and reagents.^49^ Thus, the 10 : 1 molar ratio was selected as the optimum for the esterification reaction of oleic acid with methanol using the catalyst obtained from murumuru kernel shell.

Temperature is a factor that considerably influences the conversion rates of FFA to FAME.^47^ As shown in Fig. 9d, the conversion of FFA increases considerably with increasing temperature by 61.3%, 76.7% and 97.2% at temperatures of 60 °C, 75 °C and 90 °C, respectively. At 105 °C and 120 °C, there is a decrease to 94.3% and 96.8%, respectively. The increase in the FFA conversion can not be attributed exclusively to the increase of temperature, but is also related to factors such as limited mass transfer between catalyst and reagents, and the adverse effects of methanol vaporization.^47,50^ Thus, the optimum temperature selected was 90 °C, as it is the temperature that provides adequate activation energy to protonate the carbonyl groups of the FFA, resulting in maximum conversion rate.^40^

### 3.4. Reactions with different raw materials

**Fig. 10:**
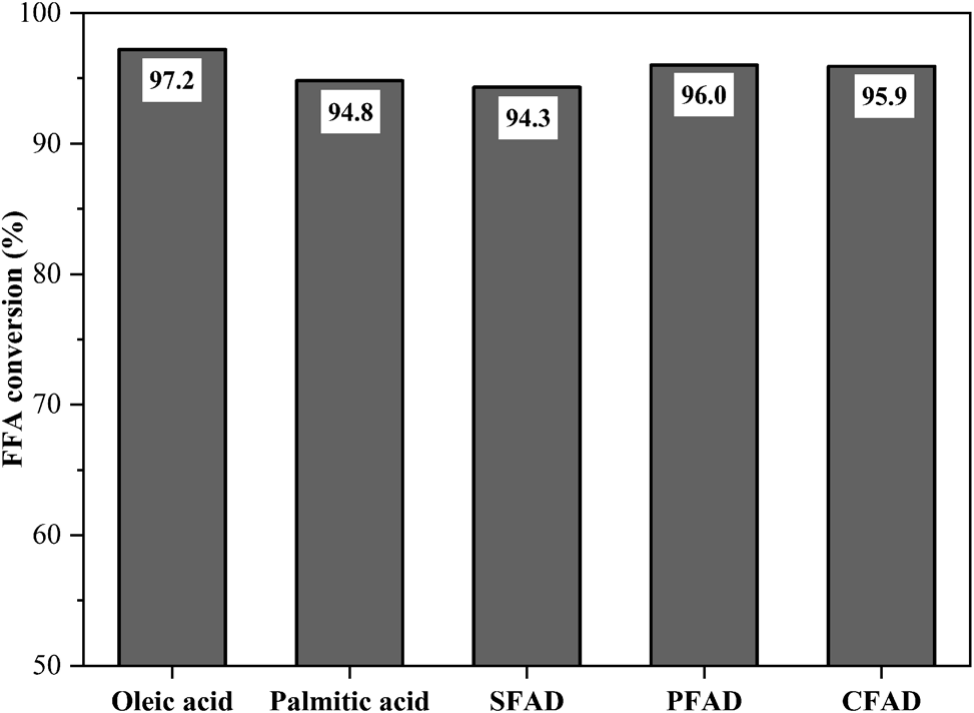
Catalyst performance on different raw materials (esterification at 90 °C for 1.5 h with the molar ratio of methanol to raw material of 10 : 1 and catalyst load of 5 wt%).

From the optimized conditions for the reaction of oleic acid with methanol – 1.5 h, 5% (w/w) of catalyst, the molar ratio of methanol to oleic acid 10 : 1 and 90 °C – it was possible to evaluate the catalyst performance against raw materials with different fatty acid compositions. The results of this investigation are illustrated in Fig. 10. The FFA conversions in the esterification reactions reached 97.2% for oleic acid, 94.8% for palmitic acid, 94.3% for SFAD, 96.0% for PFAD, and 95.9% for CFAD. Thus, the catalyst obtained from murumuru kernel shell was highly applicable to the acid raw materials used, reaching FFA conversion rates around 95% in all esterification reactions. These results indicate that its high catalytic performance is independent of the fatty acid composition of the raw material.

In addition, biodiesel samples were analyzed for ester content by gas chromatography, whose chromatograms are shown in Fig. S2.† The values determined for the biodiesel from oleic and palmitic acids were 95.9% and 93.4%, respectively. For the biodiesel from SFAD, PFAD, and CFAD, the ester content values were 92.1%, 95.2%, and 95.6%, respectively. These results show great similarities with the values determined using the titration method for the FFA conversions for the esterification reactions. This corroborates the effectiveness of using the catalyst in question for the production of low-cost and high free fatty acid biodiesel production process.

### 3.5. Biodiesel characterization

Fuel properties of biodiesel indicate the quality of the fuel and its impact on the engine. The physicochemical properties of biodiesel produced from oleic acid were determined to verify the quality of biofuel. The results of these measurements are reported in Table 1. It is observed that the produced biodiesel meets the ASTM limits.

**Table 1:** Fuel properties of biodiesel from oleic acid with ASTM standards

Fuel properties	Unit	ASTM methods	ASTM D6751	Present study
Density, at 20 °C	g cm^3^	D6890	0.875–0.900	0.887
Kinematic viscosity, at 40 °C	mm^2^ s^−1^	D445	1.9–6.0	4.65
Flash point	°C	D93	130 min	174
Cold filter plugging point	°C	D6371	Report	2
Copper strip corrosion	—	D130	3 max	1a

The density and kinematic viscosity are important fuel characteristics that influence the fuel injection operation. Higher values of these properties can negatively affect the fuel injection and lead to the formation of engine deposits.^8^ The density and kinematic viscosity of biodiesel were 0.887 g cm^−3^ and 4.65 mm^2^ s^−1^, respectively. Another important property is the flash point, which specifies the minimum temperature at which the fuel starts to ignite. The flash point determined for the biodiesel was 174 °C, higher than the minimum established by ASTM D6751. The cold property is also significant for ensuring adequate fuel storage of biodiesel and its use in winter conditions in cold countries. Thus, the determined cold filter plugging point value of 1 °C is considered excellent.

### 3.6. Catalyst reuse

**Fig. 11:**
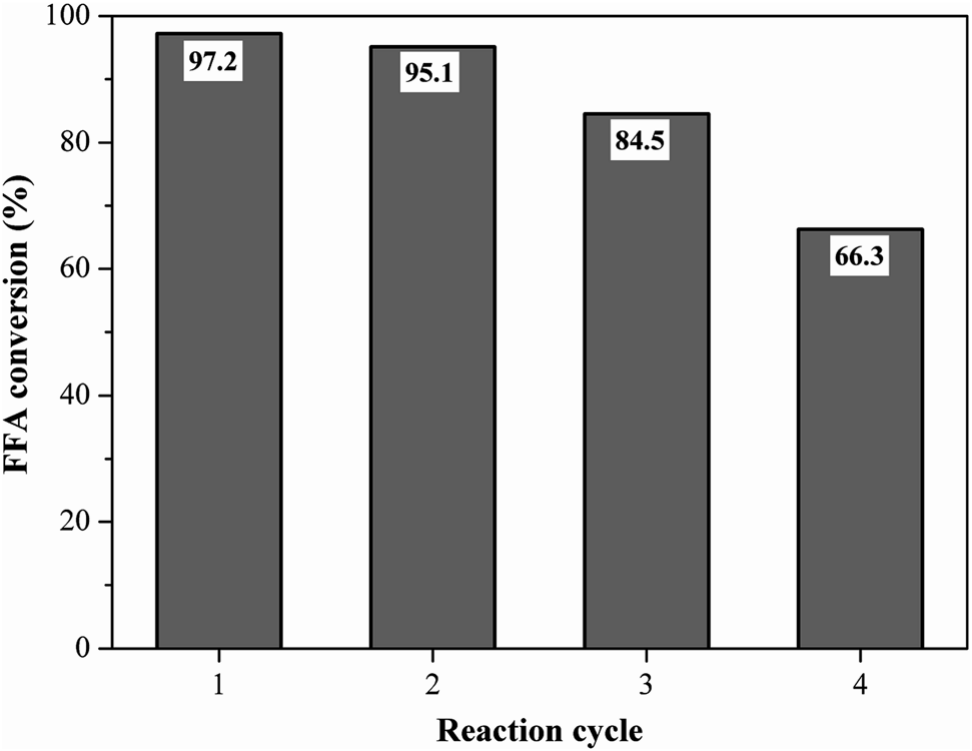
Reusability study of the catalyst (esterification at 90 °C for 1.5 h with the molar ratio of methanol to oleic acid of 10 : 1 and catalyst load of 5 wt%).

Reusability is an important property for naming the catalyst as a heterogeneous acid.^5^ The best performance catalyst selected was evaluated for its reusability under the optimized reaction conditions of oleic acid with methanol. Fig. 11 shows the fatty acid conversion values obtained in the initial reaction and reuse cycles. The catalyst remained highly efficient in its first reuse cycle, with the FFA conversion rate varying from 97.2% at the initial reaction to 95.1% (first reuse), which represents only about a 2% decrease in catalytic activity. This result shows that the catalyst was heterogeneous since the catalyst active sites were not leached in the first reuse. The second and third catalyst reuse decreased the FFA conversion to 84.5% and 66.3%, respectively, which still represent satisfactory results when compared to the literature.

Liu *et al.*^51^ prepared a carbon-based sulfonated catalyst from distillery grain, in which in the fourth reuse cycle the conversion of its catalyst fell from 97.6% to 50.7%. It is noteworthy that the catalyst can still undergo a regeneration process, *i.e.* be sulfonated again, recovering its initial characteristics. In the work of Liu *et al.*,^51^ after the regeneration process from the fourth reuse cycle, the achieved conversion was 97.9%.

The measurement of the total acid density of the resulting catalyst after the fourth reaction cycle showed a decrease from 4.2 mmol g^−1^ to 2.20 mmol g^−1^, due to the possible leaching of acidic groups, which is common from the reuse cycle in question, as reported by Lathiya, Bhatt and Maheria.^34^ In their studies with orange peel sulfonated coal, the initial acidity of the catalyst decreased from 1.5 mmol g^−1^ to 0.25 mmol g^−1^ after the fourth reaction cycle.

### 3.7. Comparison of the catalytic activity of solid sulfonated carbon-based catalysts

Table 2 shows the catalytic efficiency observed for murumuru and sulfonated biochar from different biomass materials reported in the literature, used for the esterification reaction for biodiesel production. It is possible to evaluate the catalytic performance of catalysts obtained from different carbon sources when subjected to similar synthesis processes (carbonization and sulfonation), as well as to verify the optimum reaction parameters of each catalyst in the conversion of FFA. The results obtained in the present study follow a similar trend compared to the literature data, confirming the effectiveness of the use of waste in the heterogeneous acid catalysis for biodiesel production.

**Table 2:** Biomass carbon-based sulfonated catalysts applied in esterification reaction for biodiesel production

Biomass precursor	Catalyst preparation conditions				Total acid density (mmol g^−1^)	Reaction conditions				FFA Conversion (%)	References
	Carbonization		Sulfonation			*T* (°C)	*t* (h)	Catalyst (wt%)	RM		
	Temperature (°C)	Time (h)	Temperature (°C)	Time (h)							
Murumuru kernel shell^a^	600	1	200	4	4.2	90	1.5	5	10 : 1	97.2	Present study
Cacao shell^a^	400	1	120	4	4.4	42	24	5	7 : 1	93.0	52
Cow dung^a^	500	2	180	10	16.6	90	1	4	18 : 1	96.5	53
Coffee residue^a^	600	4	200	18	0.99	60	4	5	3 : 1	71.5	28
Corn straw^a^	350	1	80	4	2.6	60	4	7	7 : 1	93.0	25
Sugar cane bagasse^a^	600	5	200	10	2.4	66	4	1	18 : 1	94.4	27
Palm kernel shell^b^	500	5	70	4	14.4	65	1	4	15 : 1	97.0	54
Bamboo^b^	500	5	70	4	8.9	65	1	4	15 : 1	95.8	54

a Sulfonation with concentrated sulfuric acid.

b Sulfonation with chlorosulfonic acid.

## 4. Conclusion

The results obtained from the investigation of the sulfonation conditions for the production of biochar from murumuru kernel shell show that the best conditions of time, temperature and solid-acid ratio are 4 h, 200 °C and 1 : 10 (w/v), respectively. Such conditions provide a heterogeneous catalyst with a total acid density of 4.2 mmol g^−1^. The reaction conditions of esterification of oleic acid with methanol showed optimal combination at 1.5 h time, 5% (w/w) of catalyst, 10 : 1 methanol to oleic acid molar ratio and 90 °C, reaching a FFA conversion of 97.2%. The high conversion values obtained in the esterification reactions reveal the promising feasibility of using agro-industrial residues as alternative precursors for the synthesis of a heterogeneous acid catalyst suitable for application in the biodiesel production process. In addition, this study emphasizes that low-cost waste acid oils are feasible sources of lipids for biodiesel production. Thus, it is possible to obtain a product with high value-added from waste raw materials by reintegrating these materials into the production process chain.

## Conflicts of interest

There are no conflicts of interest to declare.

## Supplementary Material

RA-010-D0RA03217D-s001
