# Supplementary material for: Preparation of sulfonated carbon-based catalysts from murumuru kernel shell and their performance in the esterification reaction
Source: RSC Adv. 2020 May 27;10(34):20245–56. doi: 10.1039/d0ra03217d (PMC9054225; doi:10.1039/d0ra03217d)
Supplement: RA-010-D0RA03217D-s001 [file RA-010-D0RA03217D-s001.pdf]

Electronic Supplementary Material (ESI) for RSC Advances.
This journal is © The Royal Society of Chemistry 2020

Preparation of sulfonated carbon-based catalysts from murumuru kernel shell and their performance in the esterification reaction

Ana Paula da Luz Corrêa[a], Rafael Roberto Cardoso Bastos[a], Geraldo Narciso da Rocha Filho[a], José Roberto Zamian[a], Leyvison Rafael Vieira da Conceição[a*].

[a] Federal University of Pará, Institute of Exact and Natural Sciences, Graduate in Chemistry Program, Laboratory of Catalysis and Oleochemical, Belém, Pará, CEP 66075–110, Brazil.

*Corresponding author. E-mail: rafaelvieira@ufpa.br (L.R.V. da Conceição).

Table S1. Fatty acid composition of SFAD, PFAD and CFAD.

Fatty acid	Composition (wt%)		
	SFAD	PFAD	CFAD
**Lauric (C12:0)**	-	2.4	52.6
**Myristic (C14:0)**	-	1.4	22.2
**Palmitic (C16:0)**	12.6	50.4	13.0
**Stearic (C18:0)**	3.6	4.8	12.2
**Oleic (C18:1)**	28.7	35.0	-
**Linoleic (C18:2)**	50.8	6.0	-
**Linolenic (C18:3)**	4.3	-	-
**Total Saturated**	16.2	59.0	100
**Total Unsaturated**	83.8	41.0	-

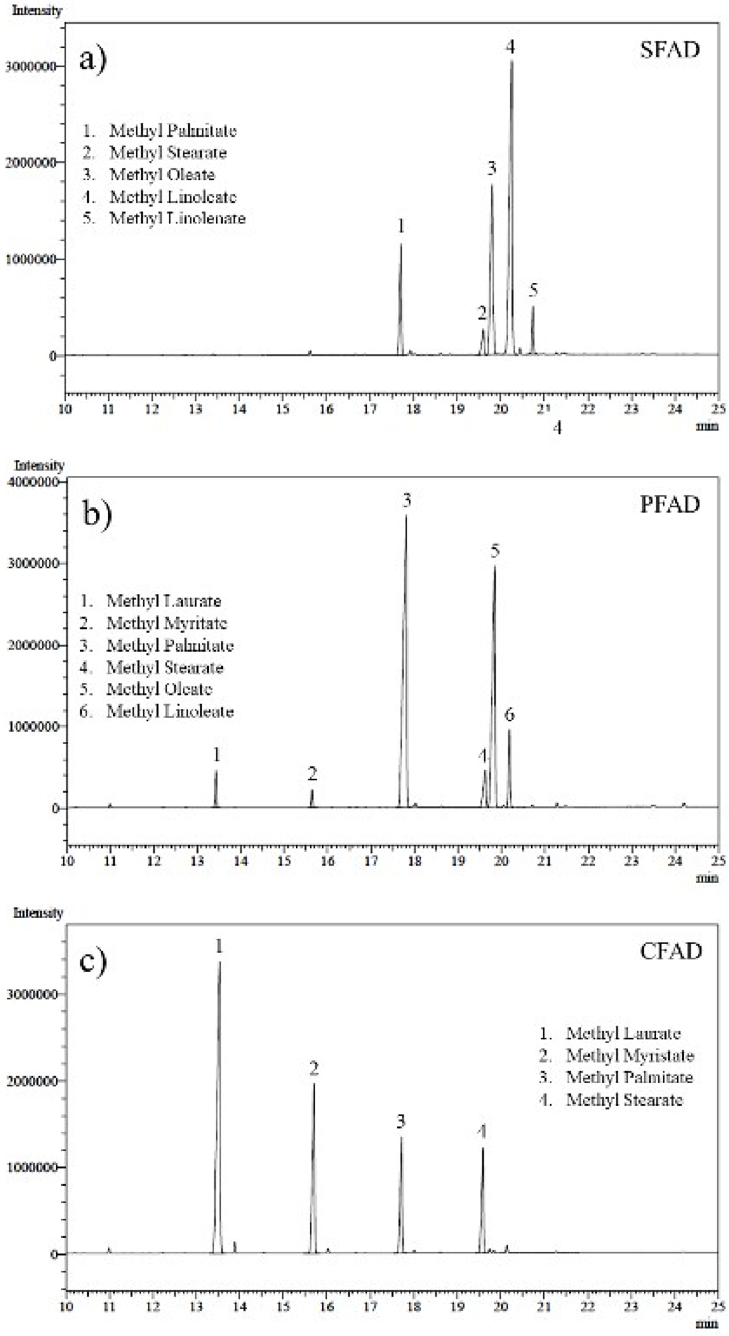

Fig. S1. Fatty acid profile of raw materials (a) SFAD, (b) PFAD and (c) CFAD.

Ester content determination

Analysis parameters

The ester contents was determined according to the European standard EN 14103, by gas chromatography using heptane as solvent and methyl heptadecanoate as internal standard in a gas chromatograph (GC) Shimadzu, model CG-2010, equipped with a flame ionization detector (FID), capillary column TG-WASMS (30 m length, 0.32 mm diameter, and 0.25 µm film). The mobile phase used was helium gas with a flow rate of 1 mL min_{-1} and an injection volume of 1 µL.

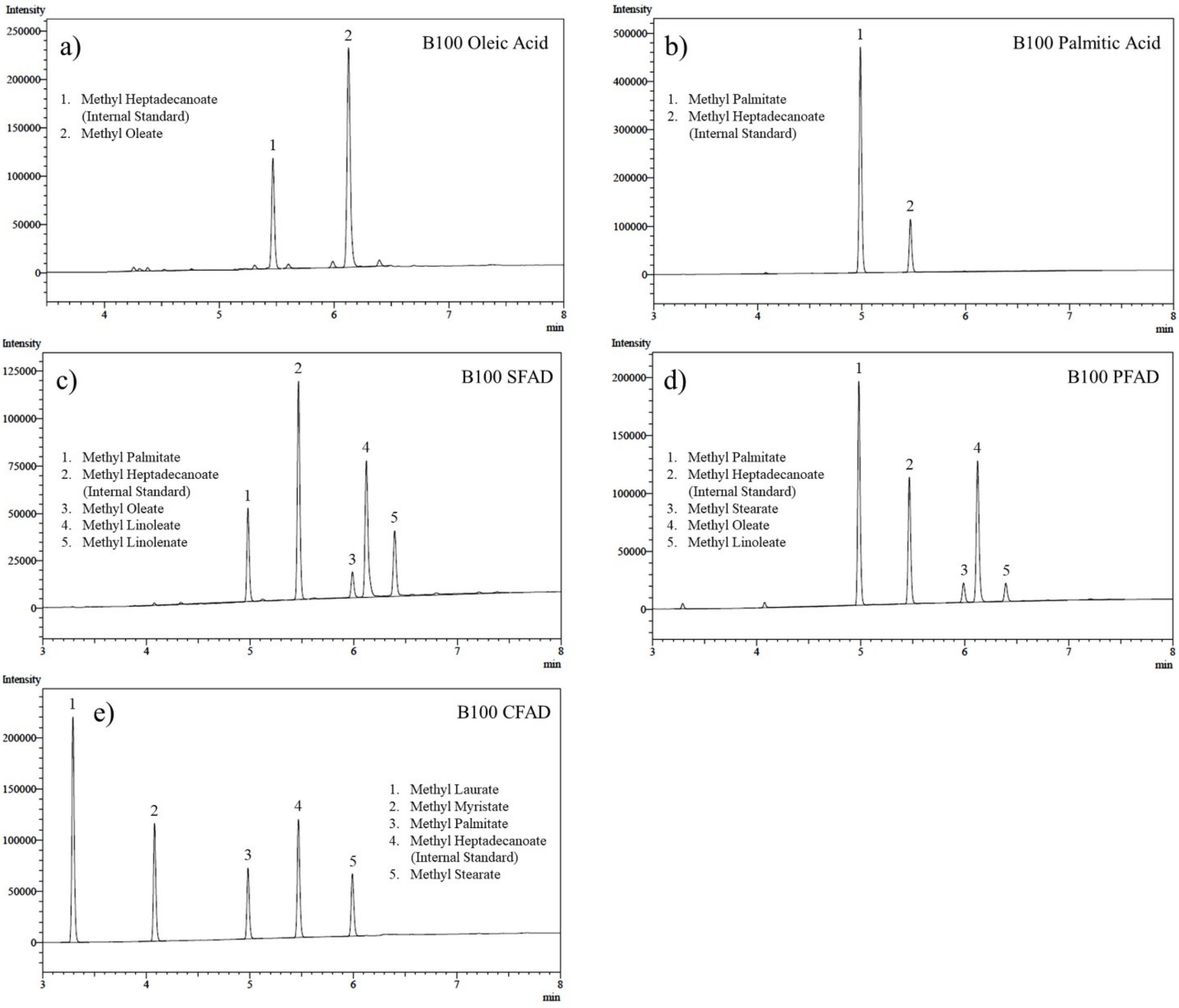

Fig. S2. Biodiesel samples chromatograms, (a) B100 oleic acid, (b) B100 Palmitic acid, (c) B100 SFAD, (d) B100 PFAD and (e) CFAD.
